# Early Second Trimester Maternal Plasma Choline and Betaine Are Related to Measures of Early Cognitive Development in Term Infants

**DOI:** 10.1371/journal.pone.0043448

**Published:** 2012-08-20

**Authors:** Brian T. F. Wu, Roger A. Dyer, D. Janette King, Kelly J. Richardson, Sheila M. Innis

**Affiliations:** Nutrition and Metabolism Research Program, Child and Family Research Institute, Department of Paediatrics, Faculty of Medicine, University of British Columbia, Vancouver, BC, Canada; Lund University Hospital, Sweden

## Abstract

**Background:**

The importance of maternal dietary choline for fetal neural development and later cognitive function has been well-documented in experimental studies. Although choline is an essential dietary nutrient for humans, evidence that low maternal choline in pregnancy impacts neurodevelopment in human infants is lacking. We determined potential associations between maternal plasma free choline and its metabolites betaine and dimethylglycine in pregnancy and infant neurodevelopment at 18 months of age.

**Methodology:**

This was a prospective study of healthy pregnant women and their full-term, single birth infants. Maternal blood was collected at 16 and 36 weeks of gestation and infant neurodevelopment was assessed at 18 months of age for 154 mother-infant pairs. Maternal plasma choline, betaine, dimethylglycine, methionine, homocysteine, cysteine, total B12, holotranscobalamin and folate were quantified. Infant neurodevelopment was evaluated using the Bayley Scales of Infant Development–III. Multivariate regression, adjusting for covariates that impact development, was used to determine the associations between maternal plasma choline, betaine and dimethylglycine and infant neurodevelopment.

**Results:**

The maternal plasma free choline at 16 and 36 weeks gestation was median (interquartile range) 6.70 (5.78–8.03) and 9.40 (8.10–11.3) µmol/L, respectively. Estimated choline intakes were (mean ±SD) 383±98.6 mg/day, and lower than the recommended 450 mg/day. Betaine intakes were 142±70.2 mg/day. Significant positive associations were found between infant cognitive test scores and maternal plasma free choline (B = 6.054, SE = 2.283, p = 0.009) and betaine (B = 7.350, SE = 1.933, p = 0.0002) at 16 weeks of gestation. Maternal folate, total B12, or holotranscobalamin were not related to infant development.

**Conclusion:**

We show that choline status in the first half of pregnancy is associated with cognitive development among healthy term gestation infants. More work is needed on the potential limitation of choline or betaine in the diets of pregnant women.

## Introduction

**Figure 1:**
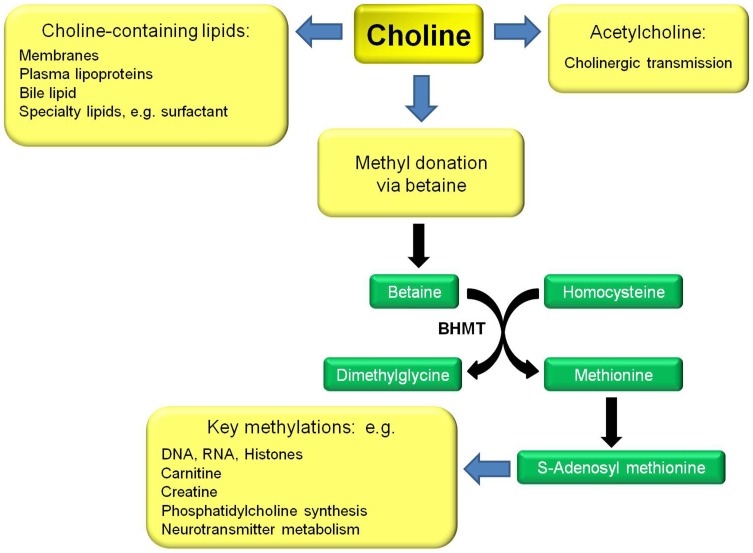
Simplified scheme to show the roles of choline and its metabolites. BHMT: betaine-homocysteine *S*-methyltransferase.

Choline (2-hydroxy-*N,N,N*-trimethylethanaminium) is an essential dietary nutrient with functions in three areas: as a source of labile one carbon units (CH_3_, methyl); as a component of lipids including phosphatidylcholine, sphingomyelin and lipid mediators such as platelet activating factor; and as a component of the neurotransmitter acetylcholine (**Figure 1**) [1], [2]. Recent interest in choline has focused on its role in neural development, with compelling evidence in rodents that maternal dietary choline deficiency in pregnancy alters fetal brain development, with effects that include decreased neural progenitor cells proliferation, increased apoptosis and global histone, DNA and gene specific hypomethylation, culminating in life-long alterations in cognitive and memory functioning [3]–[11].

**Figure 2:**
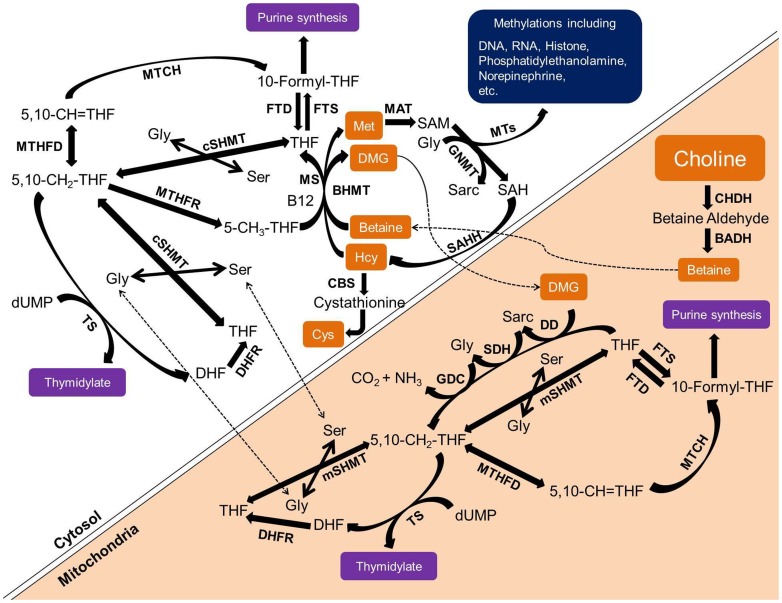
Schematic to show the role of choline in one-carbon metabolism intersecting with the methionine-homocysteine cycle. Measured metabolites are shown in orange boxes; enzymes are shown in bolded alphabets. 10-Formyl-THF: 10-formyl-tetrahydrofolate; 5,10-CH = THF: 5,10-methenyltetrahydrofolate; 5,10-CH2-THF: 5,10-methylenetetrahydrofolate; 5-CH3-THF: 5-methyltetrahydrofolate; BADH: betaine aldehyde dehydrogenase; BHMT: betaine-homocysteine *S*-methyltransferase; CBS: cystathionine beta synthase; CHDH: choline dehydrogenase; Cys: cysteine; cSHMT: cytoplasmic serine hydroxymethyltransferase; DD: dimethylglycine dehydrogenase; DHF: dihydrofolate; DHFR: dihydrofolatereductase; DMG: dimethylglycine; dUMP: 2′-deoxyuridine 5′-monophosphate; FTD: 10-formyl-tetrahydrofolate dehydrogenase; FTS: 10-formyl-tetrahydrofolate synthase; GDC: glycine decarboxylase; Gly: glycine; GNMT: glycine *N*-methyltransferase; Hcy: homocysteine; MAT, methionine adenosyltransferase; Met: methionine; MS: methionine synthase; mSHMT: mitochondrial serine hydroxymethyltransferase; MTCH: 5,10-methylenetetrahydrofolate cyclohydrolase; MTHFD: 5,10-methylenetetrahydrofolate dehydrogenase; MTHFR: 5,10-methylenetetrahydrofolate reductase; MTs, *S*-adenosyl methionine-dependent methyltransferases; SAH, *S*-adenosyl homocysteine; SAHH: *S*-adenosyl homocysteine hydrolase; SAM: *S*-adenosyl methionine; Sarc: sarcosine; SDH: sarcosine dehydrogenase; Ser: serine; THF: tetrahydrofolate; TS: thymidylate synthase. Not all enzymes and intermediates are shown in this pathway.

The role of choline as a source of methyl groups is complex and tightly inter-related with the amino acid methionine, as well as folate and vitamin B12, key vitamins which function in methyl group transfer, but require a source of methyl (**Figure 2**). In these pathways, choline is converted to betaine (*N,N,N*-trimethylglycine) which donates a methyl group to homocysteine to form methionine and dimethylglycine [2], [12]–[14]. Dimethylglycine may be further metabolized to methylglycine (sarcosine) which is then converted to glycine, with each step donating a methyl group that can be used for synthesis of methylene tetrahydrofolate, which in turn can donate a methyl group to homocysteine for synthesis of methionine in a reaction requiring vitamin B12 [15]. Methionine is the precursor of *S*-adenosyl methionine (SAM), a crucial methyl donor for numerous cellular methylations including DNA, RNA and histone methylation, conversion of norepinephrine to epinephrine, synthesis of purines and thymidylate (components of DNA and RNA), creatine (energy storage as creatine phosphate), carnitine (fatty acid transport into the mitochondria) and polyamines (cell growth), and inactivation of catecholamines [15]. Methyl transfer also forms a cycle between choline and phosphatidylcholine, since phosphatidylcholine can be synthesized from choline and diacylglycerol, or by sequential transfer of three methyl groups from SAM to phosphatidylethanolamine [16]. Major dietary sources of choline include liver, eggs and milk [17]. Betaine is also present in the diet, with rich sources being beets, spinach, quinoa, other whole grains and some shellfish [17]. A dietary need for betaine in humans has not been established.

Before birth, choline is transported across the placenta, with high concentrations of free choline in fetal plasma [18]–[20]. Although altered brain development due to deprivation of maternal dietary choline during gestation is well-established in animals [11], evidence for a similar effect in human pregnancy is lacking. One recent observational study found no association between maternal choline status during pregnancy and childhood intelligence at 5 years of age [21]. However, plasma free choline levels during the first half of gestation among pregnant women in our population overlap with the range of plasma free choline found in adults consuming choline deficient diets [22]. Since this raises the possibility of choline insufficiency, we have examined the potential associations between maternal plasma free choline and related methyl metabolites at 16 weeks of gestation and infant mental and motor skill development at 18 months of age. The primary focus was early pregnancy based on extrapolation of the critical window for long term effects of choline deprivation on brain development in rodents to humans [11], [23].

## Methods

### Subjects

This was a prospective study involving 154 healthy mother-infant pairs conducted in Vancouver, Canada. Healthy pregnant women expecting to deliver one infant with no anticipated maternal or fetal complications were enrolled at 16 weeks of gestation. The women were enrolled in a prospective study which involved investigation of the effect of the maternal status of n-3 fatty acid docosahexaenoic acid (DHA) on infant development, including intervention to increase the mothers’ DHA status [24]. Women following a vegan diet, at risk for preterm delivery, or with any known infectious or metabolic disease, were not enrolled. Infant follow-up was done only for single-birth, full-term infants (≥37 weeks gestation) with no complications likely to interfere with growth and development, or feeding. Socio-demographic information was collected. Maternal IQ was assessed using the Test of Nonverbal Intelligence, Third Edition (TONI-3), which is a non-verbal test of intelligence [25]. Usual dietary intakes were assessed using a food frequency questionnaire with the intakes of choline estimated using the United States Department of Agriculture (USDA) database on choline in foods [17]. Infant birth weight, birth length and head circumference were obtained from medical records, and information on infant feeding was recorded monthly. Infant growth was measured to 18 months of age, and weight and length were converted to z-score using the World Health Organization database [26]. The protocol was approved by the Committee for Ethical Review of Research Involving Human Subjects at the University of British Columbia and the British Columbia’s Children’s and Women’s Hospital. All mothers provided written informed consent prior to participation both for themselves and on behalf of their infants.

### Laboratory Methods

Blood samples were collected from each woman at 16 and 36 weeks of gestation; the women were requested to refrain from eating after waking until blood collection. Plasma free choline, betaine, dimethylglycine, homocysteine, methionine and cysteine were measured in plasma using isotope dilution liquid chromatography-tandem mass spectrometry (LCMS/MS), as previously described [27]. The intra- and inter- assay CVs for choline, betaine and dimethylglycine in our laboratory are 2.50% and 3.78%, 2.18% and 3.46%, and 2.42% and 3.75%, respectively. Total vitamin B12 (tB12) and holotranscobalamin (holoTC) were measured by microparticle enzyme immunoassay, and plasma folate was quantified by ion capture assay, all using an AxSym Analyzer (Abbott Laboratories, Abbott Park, IL, USA). RBC total lipids were extracted, ethanolamine phospholipids (PE) were separated, and fatty acids were analyzed using gas-liquid chromatography with flame ionization detection for assessment of maternal DHA status [28].

### Assessments of Infant Neurodevelopmental Outcome

Infant neurodevelopment was assessed at 18 months of age using the Bayley Scales of Infant Development, Third Edition (BSID-III) [29]. The BSID-III measures infant development across five domains: receptive language, expressive language, cognitive skills, fine motor and gross motor. One point was given for each successfully completed task, and the assessment continued until the infant failed five consecutive items.

### Statistical Analyses

Statistical analyses were performed using the SPSS statistical software package for Windows (version 20.0; SPSS Inc., Chicago, IL, USA).

Normality of the data was assessed using the Kolmogorov-Smirnov test. Plasma free choline, betaine and dimethylglycine were normalized with single natural log transformations. The potential associations between the measures of maternal methyl status (choline, betaine or dimethylglycine) at 16 or 36 weeks of gestation and the BSID-III infant’s cognitive, language and motor developmental raw test scores were assessed using multivariate regression. Potential confounders included in the model were maternal age, maternal IQ measured with the TONI 3, maternal ethnicity, maternal red blood cell DHA status at 16 and 36 weeks of gestation, infant breast feeding duration and infant sex. Maternal omega-3 fatty acids in pregnancy have been linked to higher scores on test of child development [30], [31], and dietary intakes of DHA and choline are positively correlated [32]. For this reason, we included the biochemical measure of maternal DHA status at both 16 and 36 weeks of gestation as covariates in the analyses. The infant characteristics of gestation length, single birth or low birth weight were not included as these were controlled for by the inclusion criteria. The present report focuses on results at 16 weeks of gestation since no associations were found between maternal plasma free choline and its metabolites at 36 weeks of gestation and infant development. Spearman correlation coefficients were used to determine the relationships among the maternal plasma free choline, betaine, dimethylglycine, methionine, homocysteine, cysteine, folate, tB12 and holoTC. All p values are based on two-sided tests, with a p<0.05 considered statistically significant.

## Results

### Associations among Methyl Metabolites and B Vitamins at 16 Weeks of Gestation

**Figure 3:**
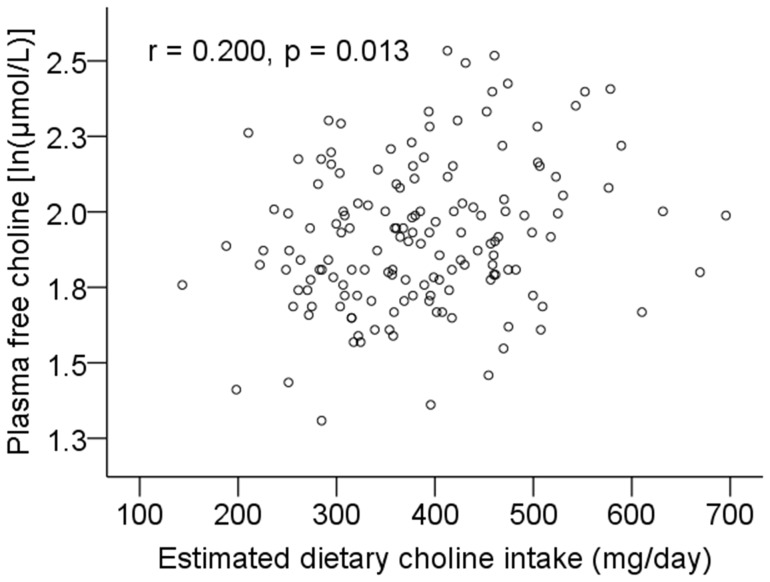
Scatter plot to show the correlation between estimated maternal choline intake and plasma free choline. The results for plasma free choline were skewed and transformed to natural log values for analysis, r = 0.200, p = 0.013.

The study population was predominantly white women (72%), with 15% of Asian background. Information on smoking and alcohol consumption were collected by self-report. Only six women reported smoking at any time during pregnancy; one stopped at 6 weeks of gestation and the remaining five women smoked <1 pack cigarettes/week. Alcohol consumption was reported at some time after conception by 47 of the women, none of whom reported more than one drink per week. All of the women reported that they had taken prenatal vitamin and mineral supplements, and none had a plasma folate <6.8 nmol/L (**Table 1**). Of the 154 women, 14 had a plasma tB12 below the lower limit of normal of 148 pmol/L, and two had a holoTC below the lower limit of normal of 35 pmol/L. HoloTC is the biologically active form of B12 and is the only form of B12 taken up and utilized by cells. All the analyses relating to infant developmental outcome were repeated excluding results for the two infants of mothers with a holoTC suggestive of vitamin B12 insufficiency, and no differences to the outcomes were found. The maternal plasma free choline showed a median of 6.70 µmol/L, with an interquartile range of 5.78 to 8.03 µmol/L at 16 weeks of gestation (Table 1). The mean ±SD for the estimated intakes of total choline and betaine were 383±98.6 mg/day and 142±70.2 mg/day, respectively, with a median (interquartile range) intake of 378 (307–457) mg/day for choline and 130 (89.9–178) mg/day for betaine. Dietary choline intake was positively correlated with the maternal plasma free choline concentration (r = 0.200, p = 0.013) at 16 weeks of gestation (**Figure 3**). There was a significant positive correlation between plasma choline and betaine, between choline and dimethylglycine, and between betaine and dimethylglycine (**Table 2**, p<0.001). Plasma methionine was also significantly and positively correlated with plasma betaine, dimethylglycine, homocysteine and cysteine, and inversely associated with tB12 and holoTC. The plasma total B12 and holoTC, but not folate, were also inversely associated with plasma homocysteine. The maternal plasma free choline, betaine and dimethylglycine at 16 weeks of gestation was significantly correlated with the same plasma measure at 36 weeks of gestation, r = 0.322, p<0.0001, r = 0.433, p<0.0001 and r = 0.524, p<0.0001, respectively. There was no significant association between the maternal plasma free choline and measures of DHA at 16 or 36 weeks of gestation, and no difference in the maternal plasma free choline, betaine or dimethylglycine among 36 week gestation women taking or not taking supplemental DHA (maternal plasma free choline: 9.75±2.34 and 9.92±2.20 µmol/L, p = 0.64; betaine: 13.0±2.61 and 13.6±2.87 µmol/L, p = 0.20; dimethylglycine: 1.37±0.57 and 1.38±0.44 µmol/L, p = 0.90 for 36 week gestation women not taking or taking supplemental DHA, respectively).

**Table 1:** Plasma methyl nutrients and metabolites, folate and vitamin B12 at 16 weeks of gestation^1^.

Plasma	Mean±SD	Median (IQR)^3^
Choline (µmol/L)	7.07±1.87	**6.70** (5.78–8.03)
Betaine (µmol/L)	13.1±3.84	**12.4** (10.4–15.1)
Dimethylglycine (µmol/L)	1.08±0.49	**1.00** (0.74–1.30)
Homocysteine (µmol/L)	**4.24±1.00**	4.10 (2.50–4.80)
Methionine (µmol/L)	**20.9±5.29**	20.1 (17.8–23.4)
Cysteine (µmol/L)	**201±29.7**	204 (185–221)
Folate (nmol/L)	36.4±8.08	**35.9** (32.3–38.4)
Total B12 (pmol/L)	**285±119**	260 (202–345)
HoloTC (pmol/L)^2^	98.6±41.7	**92.2** (71.6–118)

1 Significant skewed distributions show the median in bold, normal distributions show the mean in bold (Kolmogorov-Smirnov test, p<0.05).

2 HoloTC: Holotranscobalamin.

3 IQR: Interquartile range.

**Table 2:** Correlation coefficients among plasma methyl nutrient and metabolites, folate and vitamin B12^1^.

	Betaine	DMG^2^	Hcy^3^	Methion^4^	Cysteine	Folate	tB12^5^	HoloTC^6^
Choline	0.559*	0.467*	0.144	0.333*	0.142	0.161	0.113	0.106
Betaine	–	0.487*	−0.003	0.228*	0.122	0.116	0.064	0.083
DMG	–	–	0.157	0.301*	0.109	−0.027	0.057	−0.038
Hcy	–	–	–	0.322*	0.536*	−0.047	−0.215*	−0.219*
Methion	–	–	–	–	0.450*	−0.035	−0.013	0.001
Cysteine	–	–	–	–	–	0.101	−0.050	0.003
Folate	–	–	–	–	–	–	0.094	0.213*
tB12	–	–	–	–	–	–	–	0.657*

1 Spearman’s correlation coefficients, with significant correlations shown in bold (*p<0.05).

2 DMG: Dimethylglycine.

3 Hcy: Homocysteine.

4 Methion: Methionine.

5 tB12: Total B12.

6 HoloTC: Holotranscobalamin.

### Association between Maternal Methyl Status and Infant Developmental Outcome

**Figure 4:**
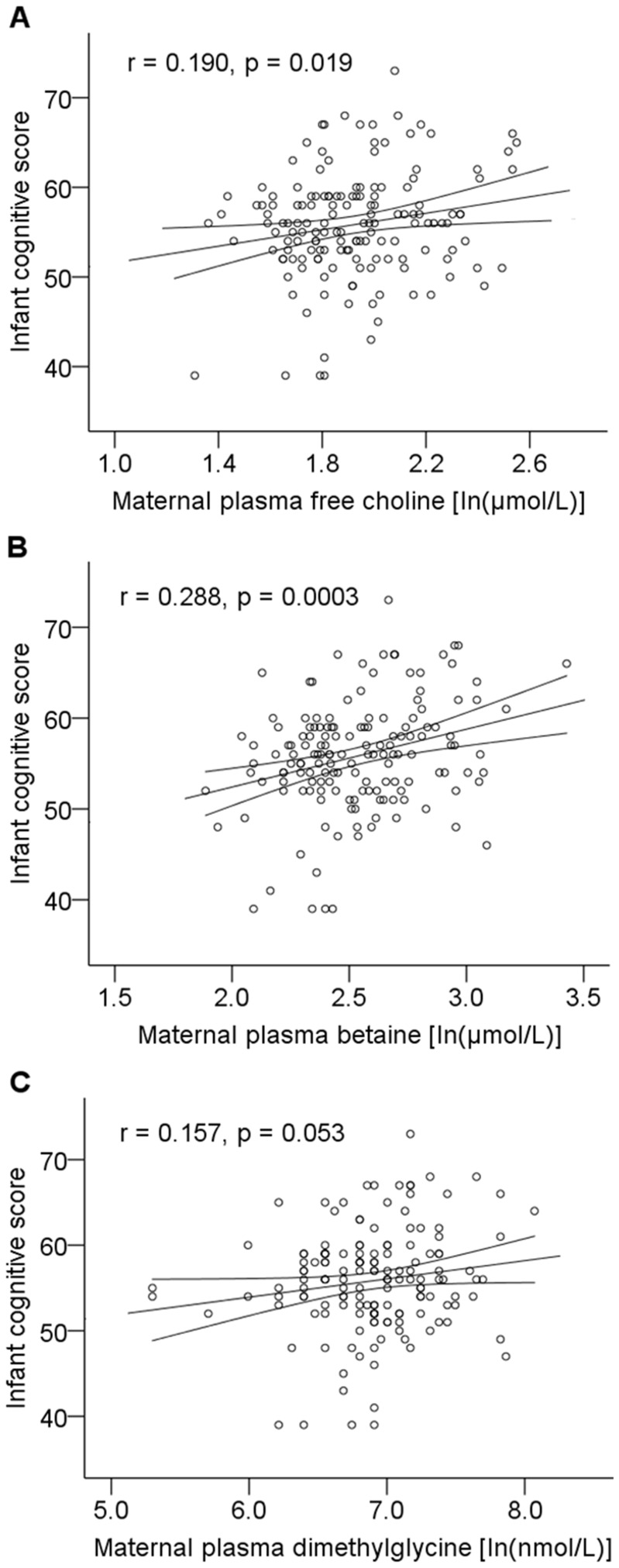
Scatter plots to show the relationship between infant cognitive scores and maternal plasma choline metabolites. Plasma metabolite concentrations were normalized by a single natural log transformation (A, B and C). Infant cognitive development was assessed using the Bayley Scales of Infant Development III. The antilog of the natural log is equivalent to the exponential of the natural log value, that is 2.718 to the exponential of the value plotted on the x axis or e^x^. The results were analysed for 154 mother-infant pairs using Pearson correlation analysis, A. choline r = 0.190, p = 0.019, B. betaine r = 0.288, p = 0.0003, C. dimethylglycine, r = 0.157, p = 0.053.

As defined by the inclusion criteria for follow-up, all of the infants were born after full-term gestation and all were single birth infants (**Table 3**). At 6 months of age, 72% of the infants were still being breast-fed. The bivariate regression analysis to address the strength of the relationship between the maternal plasma variables and the infants’ development test scores, with no consideration of confounding variables, revealed significant correlations between the maternal plasma free choline (B = 4.589, SE = 1.932, p = 0.019) and betaine (B = 6.366, SE = 1.723, p = 0.0003), and a strong trend for dimethylglycine (B = 2.134, SE = 1.095, p = 0.053) at 16 weeks of gestation and the infants’ cognitive developmental scores (**Table 4**). Scatter plots showing the bivariate associations between infant cognitive test scores and the natural log-transformed values for maternal plasma free choline (r = 0.190, p = 0.019), betaine (r = 0.288, p = 0.0003) and dimethylglycine (r = 0.157, p = 0.053) are shown in **Figure 4**. The full regression model adjusted for all variables, including the measures of maternal IQ, showed a significant positive association between the maternal plasma free choline (B = 6.054, SE = 2.283, p = 0.009) and betaine (B = 7.350, SE = 1.933, p = 0.0002) at 16 weeks of gestation and infant cognitive developmental score at 18 months of age (Table 4). Maternal IQ was used as a proxy for family income and maternal education in the final analyses; initial analyses using the latter two variables did not change the results. A trend between maternal dimethylglycine status and infant cognitive score remained (B = 2.169, SE = 1.129, p = 0.078). The adjusted multivariate regression analysis also showed a strong trend between the maternal plasma free choline (B = 2.855, SE = 1.472, p = 0.055) and betaine (B = 2.495, SE = 1.271, p = 0.052) at 16 weeks of gestation and infant gross motor development. Using the adjusted model, each 1 µmol/L increase in maternal plasma free choline, betaine and dimethylglycine at 16 weeks of gestation corresponded to an increase of 2.23, 2.70 and 0.80 in infant cognitive test score, respectively. There was no significant correlation between the maternal plasma homocysteine, methionine, cysteine, folate, tB12 or holoTC at 16 weeks of gestation, and no significant correlations between any of the maternal plasma measures at 36 weeks of gestation and infant developmental outcome.

**Table 3:** Characteristics of infants assessed at 18 months of age.

	Mean±SD	Median (IQR)^3^
Infant sex (%, boys, girls)	47,53	
Gestational age at birth (weeks)	39.6±1.16	39.9 (38.7–40.4)
Birth weight (g)	3500±476	3500 (3200–3800)
Birth length (cm)	51.8±2.32	52.0 (50.5–53.0)
Head circumference (cm)	35.1±1.42	35.0 (34.0–36.0)
Weight at 18 months (z-score)^1^	0.21±1.02	0.30 (−0.54–0.91)
Length at 18 months (z-score)^1^	0.13±1.13	0.19 (−0.68–0.97)
Head circumference at 18 months (z score)^1^	0.73±0.93	0.68 (0–1.42)
BMI at 18 months (z-score)^1^	0.22±1.03	0.19 (−0.39–0.91)
Breastfeeding at 6 months (%, yes, no)	72,28	
Receptive language score^2^	21.4±4.45	22.0 (17.3–24.0)
Expressive language score^2^	22.2±4.64	22.0 (18.0–26.0)
Cognitive score^2^	55.9±6.06	56.0 (52.0–59.0)
Fine motor score^2^	35.7±3.08	35.0 (34.0–38.0)
Gross motor score^2^	50.8±4.38	50.0 (47.0–54.0)

1 z-scores were calculated using the World Health Organization (WHO) database.

2 Assessed using the BSID-III: Bayley Scales of Infant Development, Third Edition.

3 IQR: Interquartile range.

**Table 4:** Unadjusted and adjusted regression analyses of the association between infant neurodevelopment at 18 months and maternal plasma methyl donors at 16 weeks of gestation.

	Unadjusted			Adjusted^1^		
	B^2^	SE	P	B^2^	SE	p
**Cognitive**						
Choline	**4.589**	**1.932**	**0.019** *	**6.054**	**2.283**	**0.009** *
Betaine	**6.366**	**1.723**	**0.0003** *	**7.350**	**1.933**	**0.0002** *
Dimethylglycine	2.134	1.095	0.053	2.169	1.219	0.078
**Receptive language**						
Choline	0.054	1.460	0.971	0.667	1.466	0.650
Betaine	0.291	1.315	0.825	0.309	1.247	0.804
Dimethylglycine	1.121	0.790	0.158	0.659	0.737	0.373
**Expressive language**						
Choline	−1.004	1.508	0.507	−0.509	1.667	0.760
Betaine	−1.741	1.384	0.211	−2.242	1.427	0.119
Dimethylglycine	−0.941	0.832	0.259	−1.422	0.842	0.094
**Fine motor**						
Choline	−0.198	1.017	0.846	−0.277	1.174	0.814
Betaine	0.168	0.920	0.855	0.406	1.011	0.689
Dimethylglycine	−0.289	0.558	0.606	−0.507	0.598	0.398
**Gross motor**						
Choline	1.304	1.420	0.360	2.855	1.472	0.055
Betaine	1.849	1.286	0.153	2.495	1.271	0.052
Dimethylglycine	0.874	0.786	0.268	0.829	0.767	0.281

1 Adjusted for maternal intelligent quotient, infant sex, breastfeeding duration, maternal ethnicity, maternal age, and maternal red blood cell phosphatidylethanolamine docosahexaenoic acid at 16 and 36 weeks of gestation.

2 B: Regression coefficient from the bivariate (unadjusted) or multivariate regression analysis.

* Significant level (p<0.05).

## Discussion

This study addressed the importance of choline and its metabolites betaine and dimethylglycine early in the second trimester of pregnancy on measures of child cognitive, language and motor skill development at 18 months of age. The study was confined to mother-child pairs involving only single birth infants born after full-term gestation. The findings provide evidence for an association between the mothers’ methyl status, specifically choline (p = 0.009) and betaine (p = 0.0002) in gestation and child cognitive test scores at 18 months of age, with a strong trend towards a positive association between maternal plasma free choline (p = 0.055) and betaine (p = 0.052) at 16 weeks of gestation and the infants’ gross motor development (Table 4, adjusted analyses). Studies in rats and mice have emphasized a sensitive window of maternal choline deprivation occurring between gestation days 11 and 17 which lead to morphological and molecular changes in the embryonic brain [11]. This time period in rodents corresponds to gestation beginning about 1.5 to about 3 months of gestation in human pregnancy [23], with the present study conducted at about 4 months of gestation. These results appear to be consistent with a crucial role of methylation, potentially involving synthesis of important methylated metabolites and intermediates, especially thymidylate and purines, as well as epigenetic mechanisms, such as DNA and histone methylation in early development (Figures 1 and 2) [11]. This suggestion is supported by the strong significant, positive associations between the maternal plasma free choline and betaine, choline and dimethylglycine, and choline and methionine, but not homocysteine at 16 weeks of gestation, all consistent with the importance of betaine-driven remethylation to maintain methionine for important methylation reactions. However, acetylcholine also plays a crucial role in brain development through its role in the cholinergic system [33]. Large amounts of choline are also needed to support new membrane synthesis associated with cell division and growth. Thus, the importance of choline in early brain development may be multi-factorial.

The setting of this study is on background in which the food supply has been fortified with 0.15 mg folic acid per 100 g of cornmeal or flour since 1998 [34]. In addition, all of the women in the present study reported taking prenatal multivitamin supplements, which typically contain 400 µg folic acid, variable amounts of vitamin B12, but no choline or betaine. The plasma free choline indicative of deficiency has not been defined. However, studies involving feeding a choline-deficient diet to men and post-menopausal women for 6 weeks showed a decline in plasma free choline from 9.8 to 6.8 µmol/L [22]. A substantial 56% of the women in our study had a plasma free choline concentration below 7.0 µmol/L at 16 weeks of gestation. Furthermore, 74% consumed less than 450 mg/day choline which is recommended as the adequate intake of choline for pregnant women [35]. While dietary choline intakes appear to be low in our population, other factors may influence choline status. These include several single nucleotide polymorphisms (SNP), including SNP in phosphatidylethanolamine *N*-methyltransferase (*PEMT*), choline dehydrogenase (*CHDH*) and methylenetetrahydrofolate dehydrogenase (*MTHFD1*) [36], [37], all of which may influence choline metabolism and increase sensitivity to inadequate dietary choline intakes.

Our results contradict the null findings of an association between plasma free choline in pregnancy and child IQ reported by Signore et al. [21] in the U.S. In addition to differences in the maternal and child study populations, setting, and age of child cognitive assessment, the women studied by Signore et al. had a mean plasma free choline of 9.34 µmol/L (interquartile range 7.69−11.50 µmol/L) at 16−18 weeks of gestation, with no change in plasma free choline during gestation. Women in our population appear to have a much lower choline status, with a median plasma free choline of 6.70 µmol/L at 16 weeks of gestation, which increased to a median of 9.40 µmol/L by 36 weeks of gestation. The present study also involved predominately White or Asian women, and assessed only single birth full-term gestation infants. Signore et al., on the other hand, studied women in Alabama of whom 70% were Black, and included both small for gestational age and premature infants. Relatively little, and inconsistent information is available on choline, betaine and dimethylglycine in pregnant women. Some populations have been reported to show a low plasma free choline in early gestation which increases with increasing gestation as in our studies, but others show high plasma free choline in early gestation with no change throughout gestation. Similar to our population, studies in the island of Curaçao (formerly Dutch Antilles) found a mean plasma free choline of 7.32 µmol/L at 16 weeks of gestation which increased to 10.77 µmol/L by 36 weeks of gestation [38]. Studies in Turkey, however, reported much higher plasma free choline concentrations of 14.5 and 16.5 µmol/L at 16 to 20 and 36 to 40 weeks of gestation, respectively [39]. The plasma free choline of 9.34 µmol/L among U.S. women in Alabama [21] falls between the low plasma free choline concentrations in the present study of Canadian women (Table 1), and women in Curaçao [38], and the higher plasma choline concentrations of women in Turkey [39]. It would seem important to understand the extent to which diet, genetic or other sources of variability including potential differences among laboratories in methodology to quantify plasma choline may contribute to difference in choline status among pregnant women in different countries.

In conclusion, this was an observational study that has shown dietary intakes of total choline are below current recommended intakes levels in a large (74%) proportion of pregnant women. This low intake of choline is accompanied by low plasma concentrations of free choline, lower than reported for pregnant women in other regions of the world. Plasma free choline assessed early in the second trimester of gestation was positively associated with betaine, dimethylglycine and methionine, indicating that choline is further metabolized and contributes methyl groups for regeneration of methionine, the ultimate source of methyl groups for numerous biologically important methylations. The maternal plasma free choline and betaine at 16 weeks of gestation, but not 36 weeks of gestation, were positively associated with infant cognitive development in both unadjusted and adjusted analyses.

### Limitations

This study is an observational study and no causative relationships can be drawn from our result. The estimation of dietary choline should be viewed with caution since information on choline in the Canadian food supply is unavailable, and intakes were estimated using the USDA data base on the choline content of foods. This study cohort was mostly white, educated women who breastfed their infants for six months or longer. Results from this study may not be representative of other groups of women in Canada or in other countries. The extent to which genetic variations in pathways relevant to choline metabolism impact maternal plasma free choline or betaine, their transfer to the developing infant, or maternal dietary choline need is also unknown. Although further studies to elucidate the importance of choline or its metabolite betaine in human development are needed, this study provides novel evidence that maternal methyl nutrition may play a role in early human brain development, consistent with evidence for other species.
